# Phase synchronization between culture and climate forcing

**DOI:** 10.1098/rspb.2024.0320

**Published:** 2024-06-12

**Authors:** Axel Timmermann, Abdul Wasay, Pasquale Raia

**Affiliations:** ^1^ IBS Center for Climate Physics, Busan, South Korea; ^2^ Pusan National University, Busan, South Korea; ^3^ DiSTAR, Monte Sant’Angelo, Napoli Università di Napoli Federico II, Naples, Italy

**Keywords:** climate, culture, phase synchronization, consumer-resource model, cultural evolution, planetary boundaries

## Abstract

Over the history of humankind, cultural innovations have helped improve survival and adaptation to environmental stress. This has led to an overall increase in human population size, which in turn further contributed to cumulative cultural learning. During the Anthropocene, or arguably even earlier, this positive sociodemographic feedback has caused a strong decline in important resources that, coupled with projected future transgression of planetary boundaries, may potentially reverse the long-term trend in population growth. Here, we present a simple consumer/resource model that captures the coupled dynamics of stochastic cultural learning and transmission, population growth and resource depletion in a changing environment. The idealized stochastic mathematical model simulates boom/bust cycles between low-population subsistence, high-density resource exploitation and subsequent population decline. For slow resource recovery time scales and in the absence of climate forcing, the model predicts a long-term global population collapse. Including a simplified periodic climate forcing, we find that cultural innovation and population growth can couple with climatic forcing via nonlinear phase synchronization. We discuss the relevance of this finding in the context of cultural innovation, the anthropological record and long-term future resilience of our own predatory species.

## 1. Background

Over the past ~2 million years, humans have evolved from highly specialized archaic groups in central eastern Africa (*H. habilis* and *H. ergaster*) to more versatile populations with multiple coeval species (*H. heidelbergensis*, *H. neanderthalensis*, *Denisovans* and *H. sapiens*), which managed to adapt to the colder and more volatile environments of glacial Eurasia [1,2]. This evolution was far from continuous and there is mounting evidence that climatic transitions, such as a rapid cooling event 1.126 million years ago [3], or the opening and closing of green corridors [4,5] further shaped the evolutionary trajectory of our genus *Homo*. According to this evidence, environmental conditions, as represented often by the general concept of a carrying capacity [4,6–8], could be regarded as an important agent in influencing early human survival, population growth, dispersal and adaptation. In ecological terms, the carrying capacity describes the population size that can be maintained in equilibrium for a certain area given the amount of available food resources.

Today’s situation, however, is markedly different: even though humans account only for 0.01% of all biomass on earth [9], they have been able to monopolize a large fraction of global resources [10], which in turn jeopardizes the survival of wild fauna globally [11]. As a result of our invasive, predatory exploitation of the environment, planetary boundaries are being approached at unprecedented rates or even transgressed in case of biodiversity loss, the nitrogen cycle and climate change [12–14]. Humankind is now facing the ethical dilemma that more people can presently be supported on our planet than ever before, thanks to technological advances, but at the cost of depleting resources to such an extent that living conditions for the current and future generations will be negatively impacted. How has it come to this transition from a stable pre-Holocene hunter–gatherer subsistence with little long-lasting impact on the environment to the current unprecedented and partly irreversible resource exploitation on a global scale?

Even though many aspects and stages of this transition to modern behaviour are well understood [15], it is fair to say that there is no simple conceptual model that captures the essence of combined population growth, cultural innovation and resource depletion, and that can address whether environmental (climatic) changes also played an important role in shaping cultural evolution in early humans. Modelling the dynamics of the emerging socioenvironmental crisis mathematically requires an understanding of how cultural and technological innovations translate into increased population size and how the resulting population growth will feed back onto the available resources. To this aim, we propose a simple ordinary differential equation model that can serve as a paradigmatic framework to describe different aspects of past and future technologically induced resource depletion, such as arguably megafauna extinctions in Australia or the Americas [16], or even the projected modern-day phosphorus crisis [17,18], which can impact global food production and hence human carrying capacity.

The simple stochastic ordinary differential equation model we propose describes the coupled dynamics of population and cultural growth and climatically modulated resource variability and depletion. We will describe the sensitivity of this model to intergenerational culture transfer, rates of cultural innovation, resource depletion under both constant and time-varying climatic conditions. The model is formulated to address how early hunter–gatherer populations have interacted with their environment and how the development of more advanced technologies may have helped rapid population growth and corresponding resource depletion. The model that captures the essence of some fundamental sociocultural–environmental interactions gives qualitative insights into how environmental conditions shaped cultural innovation and how learning [19,20] and the resulting growth in technological complexity and skills subsequently boosted population growth [21,22], ‘inevitably’ leading to a situation where planetary boundaries (represented in the model by the carrying capacity) will be reached.

## 2. Methods

### (a) Demographic model

**Figure 1.**
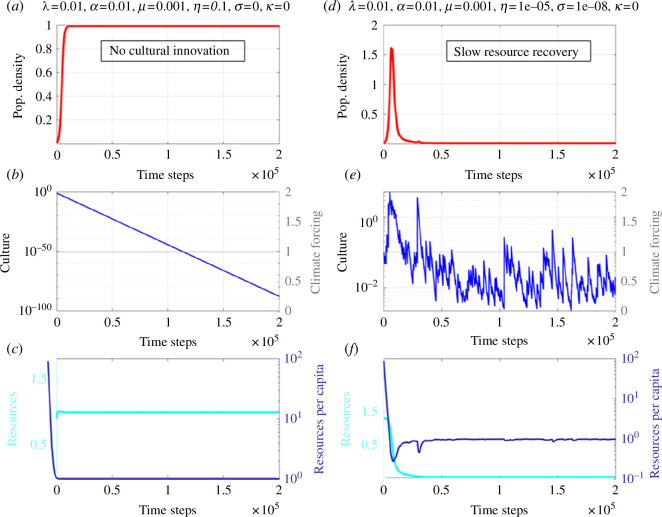
Special solutions of ecocultural model. (*a*–*c*) Numerical simulation of equations (2.1)–(2.3) for population density (red), culture (blue), carrying capacity (resources, cyan), climate forcing (grey), resources per capita (dark blue) λ=0.01,α=0.01,μ=0.001,η=0.1,σ=0 (no cultural innovation). (*d*–*f*) Same as (*a*–*c*), but for slow resource recovery and with cultural innovation: λ=0.01, α=0.01, μ=0.001, η=0.00001 (slow resource recovery), σ=0.00000001 for a specific realization of random Weibull-distributed noise.

To simulate human population density ρ in a limited region, or similarly the corresponding biomass, we start with a simple logistic equation [23–26] for the time derivate of the density,


(2.1) $$\frac{d\rho }{dt}=\alpha \rho \left[1-\frac{\rho }{K(1+c)}\right],$$

where α represents the intrinsic rate of increase of the population, c is the culture, K (in the absence of c ) the equilibrium density referred to as carrying capacity. c is introduced here as a booster to the carrying capacity [21] and K(1+c) can be considered a kind of ‘cultural carrying capacity’ [27] . Since we do not specify any region, the density ρ can be just interpreted as the total population size for our purposes. For fixed K and c (that can be interpreted as the average values in the region of interest) the model generates typical *S*-shaped sigmoid logistic curves (figure 1*a*). It is assumed here that a population with more developed culture (larger c ) can extract more food resources from the same environment (e.g. through social skills, hunting techniques or more sophisticated tools [28]), as compared to a group with less developed culture (lower *c*). Note that for populations larger than the cultural carrying capacity, the population size will decrease as the resources needed to maintain an equilibrium population at this size exceed the available resources. This leads to increased mortality until an equilibrium population size is reached after a time scale, which is controlled entirely by the demographic properties of the population, namely, α .

For the general case of time-varying and positive definite K(t)[1+c(t)] a formal general solution can be expressed as


$$\rho \left(t\right)=\frac{\rho_{0}exp\overset{t}{\int_{\text{0}}}\alpha \left(\tau \right)d\tau }{1+\rho_{0}\overset{t}{\int_{\text{0}}}\left[\alpha \left(\tau \right){\left[K\left(\tau \right)\left(1+c\left(\tau \right)\right)\right]}^{-1}exp\overset{\tau }{\int_{\text{0}}}\alpha \left(\xi \right)d\xi \right]d\tau },$$

where $\rho \left(t=0\right)=\rho_{0}$ . This solution further simplifies in case the birth rate α is assumed to be constant.

### (b) Resource depletion

The carrying capacity K (which is in the same unit as the density that is individuals per area) is a proxy for the available environmental food resources or energy. K can be modulated by climatic conditions (e.g. alternating wet–dry cycles). In its simplest form, it can be set as constant $K=K_{o}$ . However, for high population densities, there is negative feedback in which food resources can become depleted [29]. This negative feedback has been explored in the context of future population growth and human carrying capacity [30] and to understand population growth in Japan and the United Kingdom [31].

To differentiate between the demographic rebound in the presence of fixed resources and the rebound of the resources themselves, we introduce another ordinary differential equation that describes the dynamics of K. In our case, resource depletion will be expressed by a simple relaxation equation for K , which states that in the absence of a human population, the (potential) carrying capacity will reach a steady-state equilibrium $K_{o}$ with an e-folding time scale of $\eta^{-1}$ , which depends on the type of resources (e.g. plants or animals) consumed by the human population, but not the human demographic properties. As the population density grows, resources can be removed by the depletion term -μρ , which is analogous to the grazing term in predator/prey models, where ρ represents the predator and K represents the prey density. μ represents a resource depletion rate, which depends on the energy requirements of the population. The corresponding dynamical equation for the carrying capacity K then reads


(2.2) $$\frac{dK}{dt}=-\eta \left(K-K_{0}\right)-\mu \rho ,$$

where η denotes the rate of resource replenishment back to an unperturbed environmental carrying capacity $K_{0}$ , which in later sensitivity experiments will be chosen as a non-autonomous forcing $K_{0}(t)=K_{0}^{*}[1+\kappa \cos\left(\Omega t)\right]$ , to mimic the effect of temporally varying climatic conditions [32], associated with, for example, Milanković cycles [1]. The analytical solution to this equation for time-varying ρ can be formally expressed as


$$K\left(t\right)=K\left(t=0\right)+\int_{\;\;\;0}^{t}e^{-\eta \left(t-t^{\prime }\right)}\left[\eta K_{0}\left(t^{\prime }\right)-\mu \rho \left(t^{\prime }\right)\right]dt^{\prime }.$$

### (c) Modelling culture

To capture the dynamics of culture c we adopt a stochastic approach, in which the rate of change (time-derivative) of culture is given by the balance of randomly occurring innovations $\sigma \xi_{t}$ , with a standard deviation σ and stochastic variability of a given probability distribution (here chosen as a positive definite Weibull distribution with one parameter characterizing the shape and another one characterizing the scale of the probability density function) and some type of cultural information loss. We further make the assumption that innovation for early human hunter populations was already governed by cultural learning processes [33], which implies that the cultural traits (here, innovation rate) can be expressed in principle as a function of population density ρ . We can therefore represent culture accumulation as a stochastic dynamical process with a density-dependent random term,


(2.3) $$\frac{dc}{dt}=-\lambda c+\sigma \xi_{t}\rho ,$$

where λ denotes the rate of intergenerational culture loss introduced recently [34] (e.g. through lack of communication, insufficient language skills, lack of knowledge of the elders, casualties affecting the cultural innovators [33] and cultural resistance to innovation [35]), and $\sigma \xi_{t}\rho$ denotes the multiplicative knowledge gain through random innovation amplified by linear density-dependent cultural learning. Our approach differs from previous ecocultural-demographic models [21,36,37] by the fact that we describe the cultural/technological evolution by an Ornstein–Uhlenbeck process with multiplicative (density-dependent) noise [38], which to some extent mimics cultural bursting [27]. The solution of the culture equation is explicitly given as


$$c\left(t\right)=c\left(t=0\right)+\sigma \int_{\;\;\;0}^{t}\rho \left(t^{\prime }\right)\xi \left(t^{\prime }\right)e^{-\lambda \left(t-t^{\prime }\right)}dt^{\prime },$$

which illustrates that the population culture is essentially determined by the accumulation of random cultural innovations amplified by population density and evaluated within an exponentially decaying past-history kernel, with a damping time scale of about $\lambda^{-1}$ .

Our ecocultural model


$$\frac{d\rho }{dt}=\alpha \rho \;\left[1-\frac{\rho }{K\left(1+c\right)}\right]\;\frac{dc}{dt}=-\lambda c+\sigma \xi_{t}\rho \;\frac{dK}{dt}=-\eta \left(K-K_{0}\left(t\right)\right)-\mu \rho ,$$

captures the coupled dynamics of population growth, culture and resource exploitation in terms of a three-dimensional nonlinear stochastic consumer/resource ordinary different equation model. Equations (2.1) and (2.2) can also be interpreted in terms of a simple predator–prey system, which is capable of generating limit cycles or population collapses (e.g. [39]). The multiplicative stochastic culture equation (2.3) introduced here and the strong feedback between population size and culture makes the dynamics of our model more interesting and realistic, as will be demonstrated below. The parameter choice in our model is rather arbitrary, as little is known about the mathematics of cultural growth and resource depletion. Nevertheless, the rather broad parameter sweep that we will conduct for the model will still provide interesting qualitative insights into ecocultural dynamics.

In the following paragraphs, we will study the dynamics of these equations numerically with respect to the sensitivity in λ,μand σ . We have chosen to keep the original variables, rather than to non-dimensionalize the equations, because it allows for an easier and more straightforward interpretation in terms of demographic, environmental and cultural processes. The purpose of discussing the solutions for several parameter configurations is to illustrate a variety of interesting population scenarios, that can be generated by coupling a demographic equation to a simple cultural learning model and an equation capturing resource depletion and restoration.

## 3. Results

We solve the equations (2.1)–(2.3) numerically with a simple Euler-forward scheme, whose numerical stability has been tested against other methods and proven sufficiently accurate.

### (a) Numerical simulations with constant ‘climate’

We start our parameter space exploration with an idealized set-up with fixed $K_{0}=1$ (no climate forcing). In our first experiment, we exclude the cultural learning component by setting σ=0 and assume a rather small resource extraction rate ( μ=0.001 ). As expected, the population density ρ is showing the typical sigmoid evolution of the logistic equation (figure 1*a*) and saturation towards the value $\rho_{s},$ whereas the culture term decreases from its initial condition exponentially to zero (figure 1*b*). The carrying capacity K adjusts quickly (with a damping time $\eta^{-1}=10$ ) to the equilibrium value $K_{0}^{*}-\frac{\mu \rho_{s}}{\eta }∼K_{0}$ and the resource per capita value also approaches asymptotically the value 1 (figure 1*c*).

**Figure 2.**
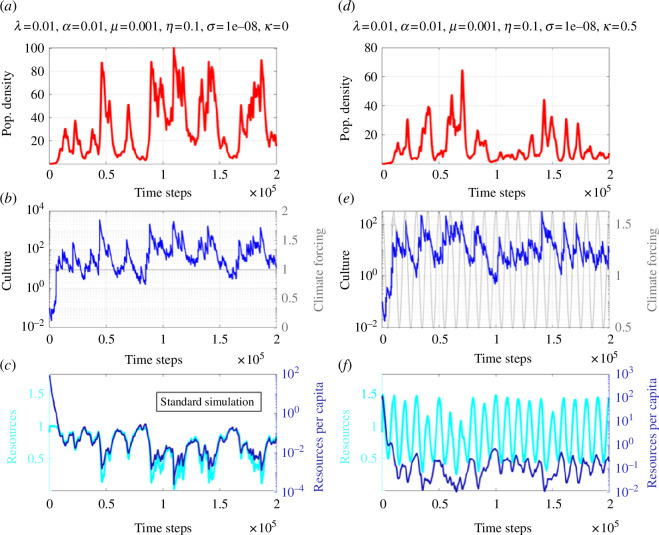
(*a*–*c*) Same as figure 1 but λ=0.01,α=0.01,μ=0.001,η=0.1,σ=0.00000001 (referred to as standard simulation). (*d–f*) Same as (*a–*c), but with periodic ‘climate’ forcing (grey) $K_{0}\left(t\right)=K_{0}^{*}\left[1+\kappa \cos\left(\Omega t\right)\right]$ with Ω=2π/10000 (standard simulation with periodic climate forcing). For panels (*c*) and (*f*), the cyan line, corresponding to the cyan *y*-axis (left), represents the time evolution of the resources, whereas the dark blue line (corresponding to the right dark blue *y*-axis) shows the resources per capita.

Introducing cultural innovation into the model changes the behaviour drastically (figure 2*a*): in the beginning (first 20 000 timesteps) accumulated cultural innovations boost population sizes to ~60 times (figure 2*a*) above the level of the simulation without culture (figure 1*a*). This illustrates the power of density-dependent stochastic learning combined with random Weibull-distributed innovations. Yet, as population size increases, more resources are extracted from the environment, and we observe a considerable drop in K(t) and resources per capita in response to the initial demographic boom, which in turn also reduces ρ (figure 2*c*). This is a crucial negative feedback, which reverses a potential exponential growth in population. With a drop in population size, the number of cultural innovations decreases (note the logarithmic abscissa for culture) and the role of cultural damping characterized by λ becomes important (figure 2*b*). As resources regrow with a time scale $\eta^{-1}$ , the system eventually resets to a state, which is similar to the initial condition and the system undergoes another stochastic boom-and-bust cycle (BBC) driven by the positive demographic-cultural feedback [34] and negative demographic-environmental feedbacks. The detailed trajectory is controlled by the randomly occurring innovations, characterized by the Weibull-distributed ξ , but a clear time scale emerges from the key parameters η,α,μ,λ and the Weibull parameters (here chosen as 1 and 0.1 for scale and shape parameters, respectively). The simulation with stochastically generated BBCs (figure 2*a–c*) will be referred to as the *standard run* and further sensitivity experiments will be compared against this baseline simulation. Note, that in our case, where BBCs emerge from the multiplicative stochastic innovation forcing. This is different from recently proposed models [32,40,41], which generate these cycles as deterministic biotic/demographic limit cycles—even without external periodic forcing.

An additional sensitivity experiment is conducted, in which we slow down the recovery of the resources K(t) to the perturbations in ρ by a factor of 10 000 $\left(\eta =10^{-5}\right)$ while maintaining cultural innovation as in the standard solution. The solution (figure 1*d–f*) shows that after the initial boom/bust episode the system collapses to a minimum population, because the resources have been exploited so quickly that they cannot regrow fast enough to support the next explosion in population and cultural innovation. Here, the resource replenishment time scale $\eta^{-1}$ is one of the key controlling factors for the simulated population collapse. This finding is important in the context of understanding planetary boundaries [13] as some resources for present-day human subsistence have already been exploited to a level beyond natural repair [14]. Our simple mathematical model illustrates that irreversible transgressions of planetary boundaries with carrying capacities dropping to near-zero values are expected to have the most detrimental long-term effect on population survival.

**Figure 3.**
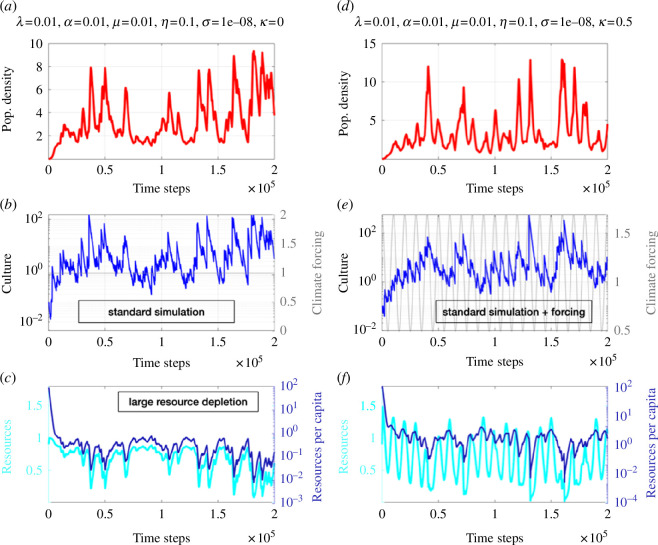
Simulations of ecocultural model. (*a*–*c*) Numerical solution of equations (2.1)–(2.3) population density, culture, carrying capacity with λ=0.01,α=0.01,μ=0.01,η=0.1,σ=0.00000001 (large resource depletion) for a specific realization of random Weibull-distributed noise. (*d*–*f*) Same as (*a*–*c*), but with periodic ‘climate’ forcing $K_{0}\left(t\right)=K_{0}^{*}\left[1+\kappa \cos\left(\Omega t\right)\right]$ with Ω=2π/10000.

In another experiment, we explore the role of the resource depletion rate μ=0.01 . Figure 3*a-c* illustrates the effect of intensifying the grazing rate. Compared with the standard simulation (figure 2*a*), we observe an increase in the frequency of BBCs, which can be explained by the fact that critically low levels of resource depletion are reached earlier, which also primes the system state earlier for the next BBC initiation. Therefore, the more intensive the resource extraction is the more unstable (in an oscillatory sense) the coupled system becomes. By comparing figure 2*a* with figure 3*a*, we can also see that for high grazing rates, the overall population stays below that of the standard run (almost by a factor of 10), which means that the maximum sustainable population level is reached earlier because the amount of food resources per capita shrinks faster (figure 3*c*). Interestingly, the temporal evolution of population density simulated in our model bears a lot of qualitative similarity to recent empirical estimates of summed probability distributions of radiocarbon dates (a proxy for changes in population size) for different regions [42].

**Figure 4.**
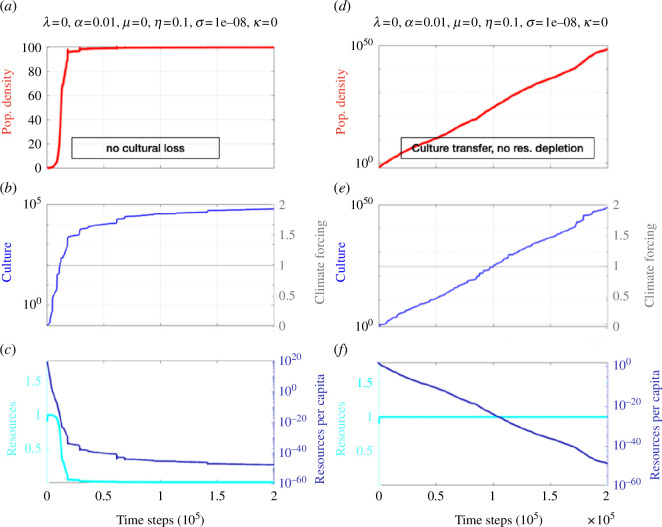
(*a*–*c*) Same as figure 3, but for λ=0, α=0.01, μ=0.001, η=0.1, σ=0.00000001 (no cultural loss) for a specific realization of random Weibull-distributed noise (*d*–*f*) Same as (figure 3*e*–*f*), but for large intergenerational knowledge transfer and without resource depletion: λ=0,α=0.01,μ=0,η=0.1,σ=0.00000001. Note the abscissa for population density is logarithmic in panel (*d*).

Moving on to another idealized case, for which we assume no culture loss owing to perfect intergenerational knowledge transfer ( λ=0 ) (figure 4*a–c*, all other parameters same as in standard run), we find that culture grows much faster, and random innovations eventually accumulate, determining the population size. Even for standard values of resource depletion and recovery, this model configuration simulates continuously decreasing resources and resources per capita (figure 4*c*). The high population density can therefore be maintained only by the extraordinarily high and monotonously increasing rates of culture (figure 4*b*) and their effect on the effective carrying capacity $K\left(1+c\right)$ . This solution is interesting from a theoretical point of view, because it allows to maintain stable populations, in spite of dwindling resources. Practically, however, if resource levels fall below a minimum level, population collapses may become imminent. Such behaviour could be implemented into the model by introducing a resource cut-off. In this case, however, the solution for this parameter configuration would qualitatively resemble figure 1*d–f*.

To further document the importance of resource limitation in long-term projections of population dynamics, we ran an idealized experiment, in which μ=0 (no resource depletion) (figure 4*d–f*). Owing to the positive feedback between density-dependent cultural learning, cultural growth and effective carrying capacity, we see an exponential growth in both culture and population size. This unrealistic solution simply illustrates the importance of resource limitation in ecocultural population dynamics.

### (b) Parameter sensitivity of ensemble mean

To further elucidate the effects of variations in intergenerational cultural loss λ and resource depletion μ on the expectation values (abbreviated as E ) of population density E[ρ] , carrying capacity E[K] and culture E[C] , we ran a 16-member ensemble of $2\times 10^{5}$ time-step-long simulation for a variety of parameter configurations. The expectation values E are calculated here as averages over both time and all 16 ensemble members.

The results in electronic supplementary material, figure S1 reveal a rather weak sensitivity of the expected culture value with respect to changes in the resource depletion rate μ , but a much stronger sensitivity with respect to the cultural loss rate λ. For λ>0.01 , cultural loss causes an overall decline in the long-term cultural expectation value and consequently in population size, as reported in indigenous populations, which suffered the loss of previously accumulated skills [33]. This, in turn, allows resources to recover, leading to an overall increase in E[K] with increasing λ (figure 3*b*). High long-term population densities can only be maintained for small resource depletion rates and minimal cultural loss (electronic supplementary material, figure S1*c*). Outside this rather narrow parameter window, populations become highly vulnerable and extinction scenarios are common.

### (c) Culture synchronizes with ‘climate’

To mimic alternating periods of abundant and sparse resources, such as determined by Milanković cycles in hydroclimate [1,43,44], we prescribe a sinusoidal forcing in the carrying capacity $K_{0}(t)$ (see chapter 2) with $\kappa =0.5,\Omega =2\frac{\pi }{10,000}$ . We refer to these highly idealized numerical experiments as ‘varying climate’ simulations (see numerical solutions in figures 2*d–f* and 3*d*–*f*.

In the absence of cultural learning, periodic forcing in the carrying capacity directly affects the population as can be seen by the corresponding analytical solution to equation (2.1) [45,46]. Given the short time scale for population growth $\alpha^{-1}=10\ll 10\;000=\frac{2\pi }{\Omega }$ , the demographic equation is in quasi-equilibrium with the slow forcing and hence we see a strong correlation between climate forcing and population density. The situation changes drastically when we also consider culture and the density-dependent innovation rate.

The numerical solution (figure 2*d–f*) with cultural learning and periodic climatic forcing shows, for the standard model parameters (figure 2*a–c*), quasi-periodic fluctuations in population size and a strong 10 000 time-step-long amplitude modulation in culture. Individual cultural innovations still occur randomly, but their overall magnitude is related to the periodically fluctuating resources. This simulation, which also exhibits a smaller amplitude of BBCs compared with the standard run (figure 2*a–c*), is interesting in the context of understanding the impact of climate on cultural evolution and interpreting transitions in the archeological record. Our simulation suggests an anticorrelation between resources per capita (dark blue curve in figure 2*f*) and culture (figure 2*e*), which phenomenologically could be (mis)interpreted as an indication that resource sparseness is the driver of cultural innovation, but in fact the underlying causality is different: more resources boost population growth (and corresponding cultural innovation) so quickly that the per capita resource term drops. The periodically forced standard simulation also reveals that not every maximum in the periodic carrying capacity is matched by maxima in population density or culture. But it appears as if some of the maxima in ρ seem to occur preferentially during certain particular phases for K . This observation, along with the fact that the overall amplitude of the periodically forced system is weaker than the BBCs in the autonomous system, is indicative of the well-known behaviour of nonlinear ordinary differential equation systems subject to non-autonomous forcings [47–49], which includes frequency entrainment, phase locking and phase synchronization [50].

To obtain a more quantitative perspective on how the ecocultural model synchronizes with the external ‘climate forcing’, we ran a 4 million-time-step-long simulation of equations (2.1)–(2.3) with periodic forcing in $K_{0}\left(t\right)$ and calculate the phase difference between forcing and response. To this end, we introduce the analytical signal approach [51], in which we extend the signal ρ(t) or c(t) in complex space as $R\left(t\right)=\rho \left(t\right)+i\rho_{H}(t)$ , where $\rho_{H}(t)$ represents the Hilbert transform of the signal (either population density or culture). We can then express the analytical signal as $R\left(t\right)=A\left(t\right)e^{i\phi \left(t\right)}$ , which allows us to calculate the phase difference δΦ between response and forcing as $\delta \Phi =\phi \left(t\right)-\Omega t$ . 1 : 1 phase synchronization occurs in a nonlinear system, when the probability distribution of δΦ (modulo 2 π ) is significantly different from a uniform distribution [50,52,53]. Here, we calculate the unwrapped δΦ for both ρ(t) and c(t) and subsequently compute the aggregated probability distribution (modulo 2π ). This analysis will reveal how external ‘climate’ forcing can impact cultural growth, whether the entrainment mechanism can be expressed in terms of the phase-synchronization concept, and which phase difference δΦ (lag) is preferred.

**Figure 5.**
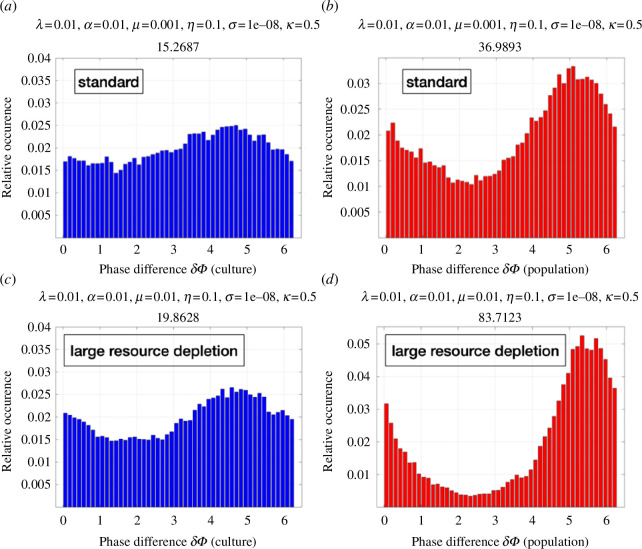
Phase synchronization quantified by the histogram of phase differences between simulated culture (blue), population density (red) and periodic climate forcing $K_{0}\left(t\right)$ for two cases: (*a,b*) standard simulation (figure 2*a–c*) and (*c*,*d*) large resource depletion (figure 3*a–c*) A uniform distribution would represent no preference for phase-angle difference (i.e. no phase synchronization). The deviation from this null hypothesis (illustrated by the relative standard deviation of the histogram expressed as percentages: 15.2, 36.9, 19.8 and 83.7) can be rejected for cases (*a*–*c*) with >99%, following the Kolmogorov–Smirnov test.

A uniform probability distribution (normalized histogram) for the phase difference between climate forcing and cultural response would indicate a case without preferred phase ‘friction’, which is a proxy for the absence of phase synchronization. For the non-autonomous standard case (figure 2*d–f*) and the large resource depletion case (figure 3*d–f*), we find clear indications of 1 : 1 phase synchronization to the periodic climate forcing (figure 5). This is most pronounced for the population density (figure 5*b*,*d*), which depicts a very tight phase coupling with the climatic forcing. The corresponding stochastic variations in culture still capture this phase synchronization, albeit with a weaker amplitude owing to the multiplicative stochastic forcing. Compared with the periodically forced standard case (figure 2*d–f*), the strong resource depletion case shows a stronger climatic phase synchronization, which can be explained by the fact that the latter exhibits a shorter frequency in the unperturbed state (figure 3*a–c*), owing to the more frequent exploitation of resources. The internal time scale of variability is now closer to the external forcing frequency which therefore makes the1 : 1 frequency synchronization more efficient in both population density and culture, as illustrated in figure 5*c,d*.

**Figure 6.**
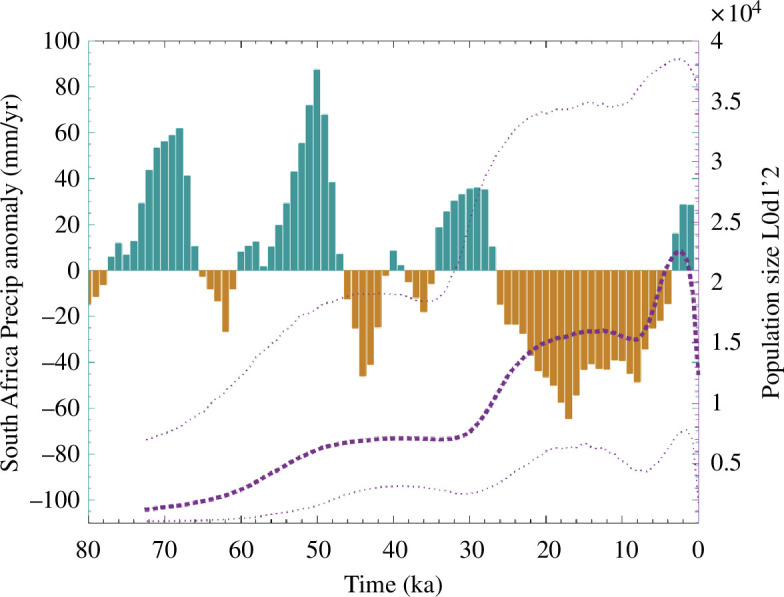
Potential evidence for climate-driven population growth: green-brown shading indicates wet–dry cycles (rainfall anomaly mm yr^−1^) in South Africa based on the Pretoria salt-pan climate reconstruction [55] and reconstructed effective population size (purple-dashed line) of L0d1’2 haplogroup (an ancestral mitochondrial lineage of modern-day Khoisan) [54] along with 95% uncertainty range (thin-dotted lines) obtained from a Bayesian skyline computation.

These results indicate that the ecocultural model can synchronize easily to external forcings via the nonlinear phase-synchronization mechanism [50]. This works particularly well, if internal consumer/resource variability, represented by the population density and resource depletion equations, respectively, exhibits similar time scales to those of the external environmental forcing. Whether this ever happened in human history remains unclear, because we know very little about the intrinsic time scales of population variability. However, recent reconstructed genetic estimates of effective population size in some ancestral southern African Khoisan populations [54] reveal fluctuations that may have occurred in unison with precessionally driven changes in hydroclimate (figure 6), with effective population growth (and genetic diversification) stalling during drier periods and increasing during wet periods in southern Africa.

Even though the actual correlation is not very high owing to the low degrees of freedom and the overwhelming trend in population size, the results are qualitatively consistent with what we would expect for the nonlinear climate phase-synchronization mechanisms proposed here and illustrated in figure 5*b,d*. Other supporting cases include the apparent link between abrupt deglacial climate change and estimates of population size on the Iberian Peninsula [56] as well as the correlation between Holocene climate change and human demography in the Rocky Mountain region [57].

In general, however, the case of climate-orchestrated culture and population growth may be difficult to ascertain. This is because imposed climate stress with impacts on human survival can lead to a multitude of responses, such as (i) habitat tracking (i.e. migration away from a region of climatic stress, towards areas of higher habitat suitability and reduced stress); (ii) cultural adaptation, in case migration is not an option or cultural innovations relevant to relieve the population from the climatic stress; which may eventually lead to (iii) selection of individuals or groups that can withstand climatic stress. The response time scales for these different processes can be quite different, which may weaken the instantaneous correlation between forcing and human reactions [57] and therefore reduce the strength of the phase synchronization.

## 4. Summary, discussion and perspectives

We introduced a simple consumer/resource model with cultural learning feedback, in which a human population is represented as a consumer that exploits limited food resources through a simple carrying capacity approach. The novel aspect of the model is the introduction of a stochastic culture equation which describes the growth of culture as a balance between cultural loss (e.g. through limited intergenerational knowledge transfer) and density-dependent multiplicative random innovations, parameterizing the process of cultural learning and cumulative cultural evolution [19,33]. In some aspects, our model shares some important characteristics with a recently published model that combines cumulative cultural evolution, ecosystem services and human population growth [37,58], and which also predicts population collapses for a wide range of parameter values, except when positive (green) technologies, which do not exhaust the natural capital, are becoming dominant.

We illustrated how culture boosts the carrying capacity [27,59] and allows more humans to survive for the given carrying capacity by increasing population density. In this regard, culture can be interpreted as influencing the efficiency with which available food resources are translated into fitness. A suite of numerical multi-parameter sensitivity experiments was conducted with the three-dimensional coupled set of stochastic ordinary differential equations. Even though we left the parameters unconstrained, the results from these simulations provide a general qualitative view of how cultural innovations may have pushed population growth in humans, which in turn activated the negative feedback associated with resource (over)exploitation and how potential climate forcing (time-varying carrying capacity) can influence population growth and subsequently culture. Our key results can be summarized as follows:

— For a wide range of parameters, the ecocultural model exhibits BBCs (e.g. figure 2*a–c*) that emerge from the negative feedback on population size owing to resource depletion and the positive feedback owing to density-dependent cultural innovation (see also [42]).
— Cultural learning plays a key role in boosting population growth and in energizing the BBCs (e.g. figure 1*a* vis-à-vis figure 2*a*). The general finding attached to these two points is that culture may drive the emergence of a typical *r*-strategist (boom-and-bust) population dynamics [60] in an otherwise *K*-strategist species because of rapid resource overexploitation. Since this dynamic is more likely to take place with little diverse resources, it may have had an important role in past human population crashes.
— The time scale characterizing resource recovery is an important parameter to determine whether a long-term population collapse is imminent or whether the system stabilizes (see e.g. figure 1*d*). This is key to understand the resilience of populations to past and future climate change. Exploiting resources beyond recovery (e.g. for the phosphorus cycle, freshwater or biodiversity) can accelerate the emergence of irreversible transitions in the biophysical earth system, as stated by the planetary boundaries concept [13]. This may exacerbate the risk of food insecurity, for instance, creating stress on individual people, societies and countries. In worst cases, this can lead to migration [61], political tensions and possibly also wars, which in turn can further limit food supplies [62,63].
— Non-autonomous forcing [32] introduced in the relaxation term of the resource equation to mimic climate variability (e.g. Milanković cycles) can synchronize both population density and to a lesser degree cultural development (figure 5). This latter point is crucial to understand which set of conditions, in terms of climate change and population density, most likely fostered major cultural innovations.

Even though our model is not suitable yet to quantitatively assess the emergence of past cultural innovations, it can nevertheless provide a conceptual, albeit non-spatial, framework to qualitatively understand the interaction between changing environments and population growth for socially active agents. Additional research with inverse modelling techniques [64] may help to further constrain some of the parameters of the model using empirical data, such as population size estimates, climate time series and cultural information. Our model may also find applications in linking past climate shifts with cultural innovations or increases in population numbers, as illustrated in figure 6. The rapid transitions from one neolithic tool tradition to the next and the associated boosting in tool complexity and variety are good examples that document increasing human resilience and cultural carrying capacities, as represented also by the expansion of the human climate niche and the corresponding geographic ranges [1,2,65]. Whether these innovations emerged in response to climatic shifts, or whether tools simply allowed hominins to expand their climate niche is unclear.

Our model also captures the process of resource depletion. Where and when early human populations experienced for the first time self-inflicted large-scale reduction in food resources, with potential impacts even on climate is still debated [66,67]. Megafauna extinctions in Australia and the Americas, which accelerated shortly after *H. sapiens* began to populate these regions [16], may have created initial food scarcity and insecurity; but the successful peopling of these continents from 40 000 to 8000 years ago is testimony to the fact that cultural adaptation (e.g. acquisition of improved hunting techniques and toolmaking proficiency) may have helped overcome potential phases of anthropogenic resource depletion.

The model presented here is highly idealized and it does not account for spatial dispersal; yet it captures some qualitative features of recent estimates of Holocene population size variations in the Middle East, Europe and South America quite well [42] (see their fig. 1). Our model assumes that individuals belong to a single population, and that culture and carrying capacity can be treated as spatially averaged quantities within a limited region. Despite these limitations, it captures interesting ecocultural interactions, and we hope that it stimulates further research into population BBCs, past, present and future resource depletion, planetary boundaries and the role of stochastic cultural learning in population dynamics.

## Data Availability

The Matlab code used to conduct the simulations and create the figures is available on Dryad [68]. Electronic supplementary material is available online at Figshare [69].
